# Local intra-articular injection of rapamycin delays articular cartilage degeneration in a murine model of osteoarthritis

**DOI:** 10.1186/s13075-014-0482-4

**Published:** 2014-11-17

**Authors:** Koji Takayama, Yohei Kawakami, Makoto Kobayashi, Nick Greco, James H Cummins, Takehiko Matsushita, Ryosuke Kuroda, Masahiro Kurosaka, Freddie H Fu, Johnny Huard

**Affiliations:** Stem Cell Research Center, University of Pittsburgh, Pittsburgh, PA 15219 USA; Department of Orthopaedic Surgery, University of Pittsburgh, Pittsburgh, PA 15260 USA; Department of Orthopaedic Surgery, Kobe University Graduate School of Medicine, Kobe, 650-0017 Japan; Stem Cell Research Center, Department of Orthopaedic Surgery, University of Pittsburgh, School of medicine, 450 Technology Drive, Bridgeside Point II, Suite 206, Pittsburgh, PA 15219 USA

## Abstract

**Introduction:**

Recent studies have revealed that rapamycin activates autophagy in human chondrocytes preventing the development of osteoarthritis (OA) like changes *in vitro*, while the systemic injection of rapamycin reduces the severity of experimental osteoarthritis in a murine model of OA *in vivo*. Since the systemic use of rapamycin is associated with numerous side effects, the goal of the current study was to examine the beneficial effect of local intra-articular injection of rapamycin in a murine model of OA and to elucidate the mechanism of action of rapamycin on articular cartilage.

**Methods:**

Destabilization of the medial meniscus (DMM) was performed on 10-week-old male mice to induce OA. Intra-articular injections of 10 μl of rapamycin (10 μM) were administered twice weekly for 8 weeks. Articular cartilage damage was analyzed by histology using a semi-quantitative scoring system at 8 and 12 weeks after surgery. Mammalian target of rapamycin (mTOR), light chain 3 (LC3), vascular endothelial growth factor (VEGF), collagen, type X alpha 1 (COL10A1), and matrix metallopeptidase 13 (MMP13) expressions were analyzed by immunohistochemistry. VEGF, COL10A1, and MMP13 expressions were further examined via quantitative RT-PCR (qPCR).

**Results:**

Intra-articular injection of rapamycin significantly reduced the severity of articular cartilage degradation at 8 and 12 weeks after DMM surgery. A reduction in mTOR expression and the activation of LC3 (an autophagy marker) in the chondrocytes was observed in the rapamycin treated mice. Rapamycin treatment also reduced VEGF, COL10A1, and MMP13 expressions at 8 and 12 weeks after DMM surgery.

**Conclusion:**

These results demonstrate that the intra-articular injection of rapamycin could reduce mTOR expression, leading to a delay in articular cartilage degradation in our OA murine model. Our observations suggest that local intra-articular injection of rapamycin could represent a potential therapeutic approach to prevent OA.

## Introduction

Osteoarthritis (OA) is the most common disorder of the joint, causing joint pain and dysfunction in affected patients. Multiple factors have been suggested in the pathogenesis of OA, including mechanical, genetic, and aging-associated factors. At the cellular level, OA is characterized by a loss of tissue cellularity and extracellular matrix (ECM) damage [1]. Chondrocytes are the resident cells found in cartilage tissue and are responsible for both synthesis and turnover of the ECM [2]; therefore, maintaining the health of chondrocytes is an important factor for preventing articular cartilage degeneration.

Autophagy is a cellular self-digestion process which is evolutionally observed among species and generally activated under conditions of nutrient deprivation. When cells experience nutrient deprivation, they maintain only the minimum amount of essential components in order to prevent energy loss, thereby degrading unnecessary intracellular components through the process of autophagy. Thus, autophagy is perceived as an important mechanism for cell survival when exposed to various stresses [3]. Recently, a growing number of studies have revealed that autophagy also plays an important role in physiological housekeeping processes through the intracellular clearance of unnecessary proteins, pathogens, and damaged organelles, which include mitochondria, peroxisomes, and the endoplasmic reticulum (ER) [4-6]. These functions have important implications in the pathogenesis of a variety of human diseases, such as neurodegenerative, cardiac, muscular, and inflammatory diseases [4]. One of the common pathological discoveries in such diseases is the accumulation of aggregate-prone proteins, potentially harmful to the cells, which likely occur due to an impairment of autophagy [7]. Autophagy has, therefore, been suggested to play a preventive role in the development of particular human diseases. Based on the broad cell-protective functions of autophagy, it is plausible that autophagy play a protective role in chondrocytes under stresses, and may prevent the OA degeneration of chondrocytes.

Recent studies have revealed that rapamycin activates autophagy in human chondrocytes *in vitro*, which acts to prevent the expression of OA-like changes [8]. Similarly, the systemic injection of rapamycin has been shown to reduce the severity of OA in an experimental murine model [9]. Clinically, rapamycin is currently used as an immunosuppressant in organ transplantation patients. Clinical studies have shown that rapamycin is not only able to provide a low rate of acute rejection and significant improvement in kidney allograft function, but it can also significantly decrease the incidence of post-transplant malignancies [10,11]; however, the systemic use of rapamycin is associated with many side effects, including increases in serum cholesterol and triglycerides, anemia, proteinuria, skin rashes, delayed wound healing, and diarrhea, which can eventually lead to rapamycin withdrawal [12].

Due to these severe side effects, the local intra-articular injection of rapamycin may be more appropriate for clinical use than systemic administration. However, it is unknown whether the intra-articular injection of rapamycin can affect the degeneration of articular cartilage. The objective of this study was to examine the effect of local intra-articular injection of rapamycin on articular cartilage in a murine model of OA. Our observations suggest that intra-articular injection of rapamycin protects articular cartilage from osteoarthritic changes, and may represent an effective and safe therapeutic delivery method to prevent articular cartilage degeneration.

## Methods

### Surgical induction of osteoarthritis in mice

All studies were performed according to protocols approved by the University of Pittsburgh’s Institutional Animal Care and Use Committee. Forty, 10-week-old, male C57Bl/6 J mice were used in this study. The animals were anesthetized with 3% isoflurane in O_2_ gas (1.5 liter/minute). Experimental osteoarthritis was induced by destabilizing the medial meniscus (DMM) in the right knee [13]. Briefly, the joint capsule was opened with an incision just medial to the patellar tendon and the medial meniscotibial ligament was sectioned with microsurgical scissors. As a control, surgery was performed on left knee joints but the ligaments were left intact and used as sham joints. All mice were allowed to move freely within their cages after surgery. After surgical induction of OA, the animals were divided into two groups, namely the rapamycin treatment group and the dimethyl sulphoxide (DMSO) (control) group. Mice were sacrificed at 8 and 12 weeks after DMM surgery and subjected to histological and gene expression analyses.

### Rapamycin treatment

To perform the intra-articular injection, the animals were first anesthetized and the skin was subsequently incised longitudinally over the center of the knee joint. The capsule and patellar tendon were exposed to clarify the anatomy of the knee to ensure reproducible intra-articular injection of either rapamycin or DMSO (control). Rapamycin was obtained from LC Laboratories (Woburn, MA, USA) and was dissolved in dimethyl sulphoxide (DMSO) to make a 50 mg/ml stock solution. For injection, the stock solution was diluted in PBS: 10 μl of rapamycin (10 μM) with DMSO (0.02%) was administered twice a week for 8 weeks in both knees in the rapamycin group. The dosage and frequency of rapamycin was selected based on previous studies [8,14]. These studies have demonstrated that autophagy activation by 10 μM rapamycin regulated the changes in the expression of OA-related genes through the modulation of apoptosis and reactive oxygen species (ROS) in human chondrocytes *in vitro* [8], and the effect of rapamycin on mechanical-injury-induced cell death continued up to 96 hours after treatment in *ex vivo* study [14]. The control group received intra-articular injections of 10 μl of DMSO (0.02%) in both knees according to the same schedule as the rapamycin group.

### Histological evaluation for articular cartilage degeneration

Six knee joints from each group were fixed in 10% neutral buffered formalin, decalcified with 10% formic acid, and embedded in paraffin. Coronal histological sections were performed through the joint at 80-μm intervals and stained with toluidine blue, and articular cartilage damage was scored by two observers blinded to sample identity using a scoring system reported by Glasson [15]. In this system, histological scores were measured in four quadrants (medial femoral condyle, medial tibial plateau, lateral femoral condyle, and lateral tibial plateau). The scores are defined as follows: 0: normal; 0.5: loss of toluidine blue without structural changes; 1: small fibrillations without loss of cartilage; 2: vertical clefts extending from the articular surface down to the layer immediately below the superficial tangential zone with some loss of surface lamina; 3: vertical clefts/erosion extending down to the calcified articular cartilage comprising <25% of the quadrant width; 4: vertical clefts/erosion extending down to the articular calcified cartilage comprising 25 to 50% of the quadrant width; 5: vertical clefts/erosion extending down to the calcified articular cartilage comprising 50 to 75% of the quadrant; and 6: vertical clefts/erosion extending down to the calcified articular cartilage comprising >75% of the quadrant width. Ten sections of each knee joint were scored and the final score was expressed as the summed histologic score for each joint. The summed score represents the additive score of each quadrant of the joint on each histologic section through the joint. This method of analysis enabled assessment of the severity of the lesions while also reflecting the surface area of articular cartilage affected with OA lesions.

### Immunohistochemistry

We performed immunohistochemical staining to detect the expression of phosphor-mammalian target of rapamycin (p-mTOR), light chain 3 (LC3), vascular endothelial growth factor (VEGF), collagen, type X alpha 1 (COL10A1), and matrix metallopeptidase 13 (MMP13) in the articular cartilage. Rabbit polyclonal antibodies to p-mTOR, LC3 (Cell Signaling, Beverly, MA, USA), VEGF, MMP13 (Abcam, Cambridge, MA, USA), and COL10A1 (Abbiotec, San Diego, CA, USA) were used at a 1:100 dilution and incubated overnight at 4°C. Alexa Fluor 488-conjugated donkey anti-rabbit IgG (Molecular Probes, Grand Island, NY, USA) was used at 1:200 dilution as the secondary antibody against the primary antibodies at room temperature for 2 hours. After staining, we evaluated the expression levels in the articular cartilage using Northern Eclipse software (Empix Imaging Inc, Cheektowaga, NY, USA).

### RNA isolation and quantitative RT-PCR (qPCR)

Four knee joint specimens from both the control and experimental group were used for qPCR. Total RNA was extracted from cartilage using TRIzol reagent (Invitrogen, Carlsbad, CA, USA) with QIAshredder homogenizers and the RNeasy Mini kit (Qiagen, Hilden, Germanay) according to the manufacturer’s protocol. One microgram of total RNA was used for random hexamer-primed cDNA synthesis using the SuperScript II pre-amplification system (Invitrogen, Carlsbad, CA,USA). Quantitative RT-PCR reactions were performed in triplicate using iQ5 (Bio-Rad, Hercules, CA, USA) with Maxima SYBR Green/ROX qPCR Master Mix (Thermo, Thermo Scientific, Rockford, IL, USA) and 300 nM of each primer. The primers were designed based on the sequences in the GenBank database. The primer pairs used for this study are shown in Table 1. Specificity of the reactions was confirmed by 2.5% agarose gel electrophoresis.

**Table 1:** **Primer sequences and product side for real-time PCR**

**Gene**		**5′ DNA sequence 3′**	**Amplicon length, base pairs**
*VEGF*	Forward	CCCACGTCAGAGAGCAACA	101
	Reverse	TCACATCTGCTGTGCTGTAGG	
*Col10*	Forward	AGGCAAGCCAGGCTATGGAA	83
	Reverse	GCTTCCCCGTGGCTGATATTC	
*MMP13*	Forward	GCTGCGGTTCACTTTGAGAA	106
	Reverse	GGCGGGGATAATCTTTGTCCA	

### Statistical analysis

All the data were expressed as the mean ± SD. The Mann-Whitney *U*-test was used for direct comparisons between the two groups. One-way analysis of variance (ANOVA) or the Kruskal-Wallis test was applied for multiple comparisons between independent groups. Pairwise, multiple comparisons were performed using the Tukey-Kramer or Scheffé post hoc test. Data analyses were performed using PASW Statistics 21 (SPSS, Chicago, IL, USA). Statistical significance was determined at level of *P* <0.05.

## Results

### The local intra-articular injection of rapamycin delayed articular cartilage degradation

**Figure 1:**
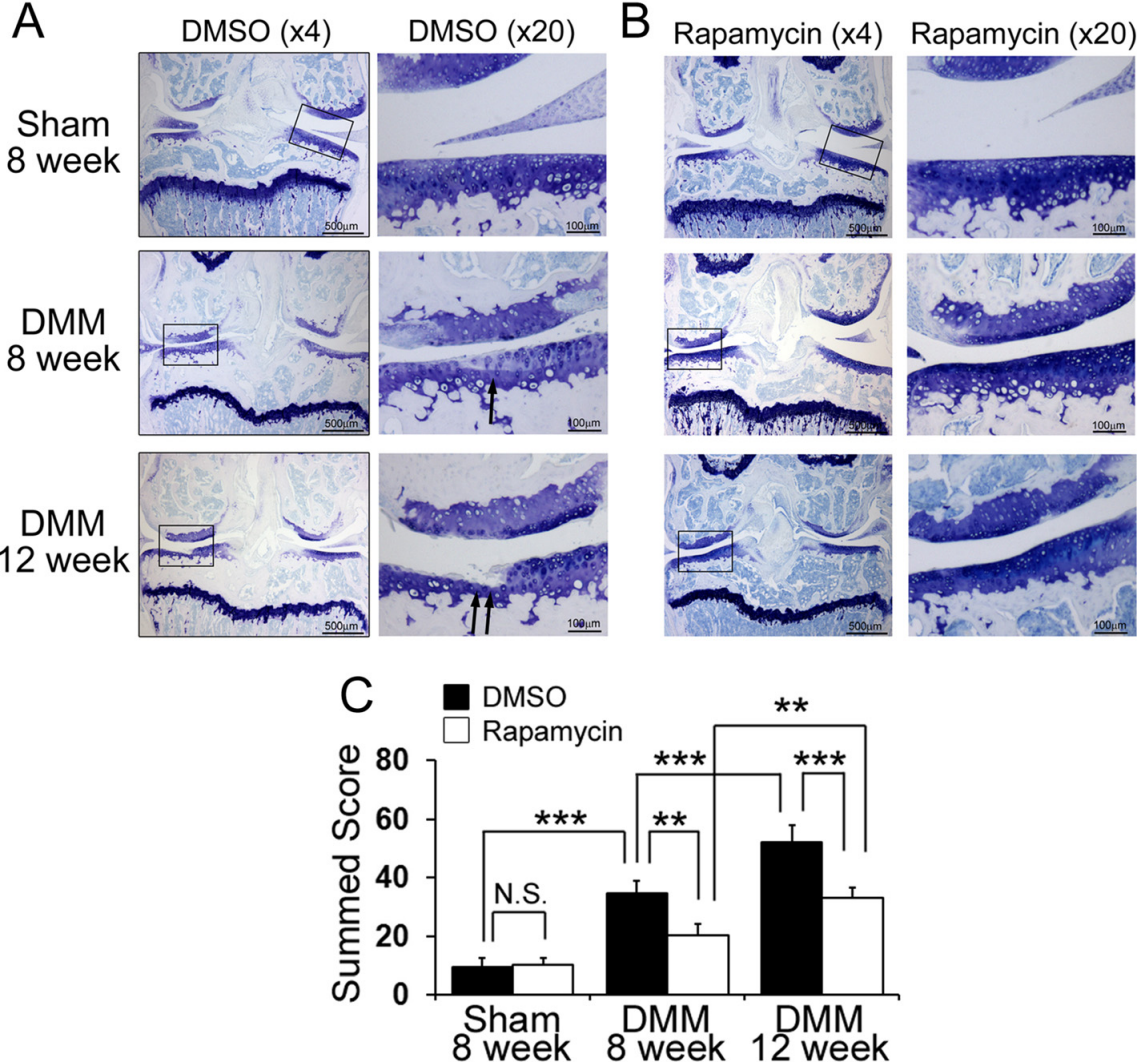
**Histological evaluation of osteoarthritis (OA). (A, B)** Representative images of toluidine blue staining from dimethyl sulphoxide (DMSO) **(A)** and rapamycin **(B)** treated mice at 8 and 12 weeks after destabilization of the medial meniscus (DMM) or sham surgery. Boxed areas in the toluidine blue stained image at the left side (x4) indicate the regions shown in the enlarged toluidine blue stained area at the right side (x20). Scale bar = 500 μm (x4) and 100 μm (x10). Arrows indicate the osteoarthritic change after DMM surgery. **(C)** Graph indicating the summed OA scores. Summed OA scores were calculated from all four quadrants and eight sections from each knee. ***P <0.001, **P <0.01.

There were no structural changes at the anterior cruciate ligament or meniscus identified in the sham knees that were treated with rapamycin or DMSO. Side effects such as weight loss, skin rashes, delayed wound healing, or diarrhea were not observed after local intra-articular injection of rapamycin. Histological sections demonstrated significantly less articular cartilage degeneration in the experimental group treated with local intra-articular injections of rapamycin at 8 and 12 weeks after induction of OA with DMM surgery compared to the OA-induced mice treated with DMSO. Histological grading showed that the DMSO-treated mice had a loss of proteoglycan staining with articular fibrillation at 8 weeks and losses of hyaline cartilage, proteoglycan staining, and lesions extending into the calcified cartilage at 12 weeks after surgery (Figure 1A). In contrast, rapamycin-treated mice showed a focal loss of proteoglycan staining without severe articular cartilage loss at 8 weeks and lesions with a loss of proteoglycan staining slightly increased 12 weeks after surgery; however, hyaline cartilage was preserved (Figure 1B). The extent of OA was evaluated by scoring specific parameters of OA, and is presented as summed scores (the higher the score the greater the articular cartilage degeneration) (Figure 1C, Table 2). Using the summed OA score, DMSO-treated mice developed OA in a time-dependent manner and had a significantly higher score than the rapamycin-treated mice at 8 and 12 weeks following DMM surgery (Figure 1C, *P* = 0.001 at 8 weeks and *P* <0.001 at 12 weeks). The summed OA score in the rapamycin-treated mice at 12 weeks was increased compared to the score at 8 weeks (Figure 1C, *P* <0.001). These results suggested that the intra-articular injection of rapamycin did not completely prevent, but delayed articular cartilage degradation in the presence of meniscus injury after DMM surgery.

**Table 2:** **Summed score**

**Summed score**		
Sham 8 weeks	DMSO	9.7 (SD 2.9)
	Rapamycin	10.2 (SD 2.5)
DMM 8 weeks	DMSO	34.7 (SD 4.5)
	Rapamycin	20.5 (SD 3.6)
DMM 12 weeks	DMSO	52.2 (SD 6.0)
	Rapamycin	33.3 (SD 3.3)

DMM, destabilization of the medial meniscus; DMSO, dimethyl sulphoxide.

### The local intra-articular injection of rapamycin decreased p-mTOR and increased LC3 expression

**Figure 2:**
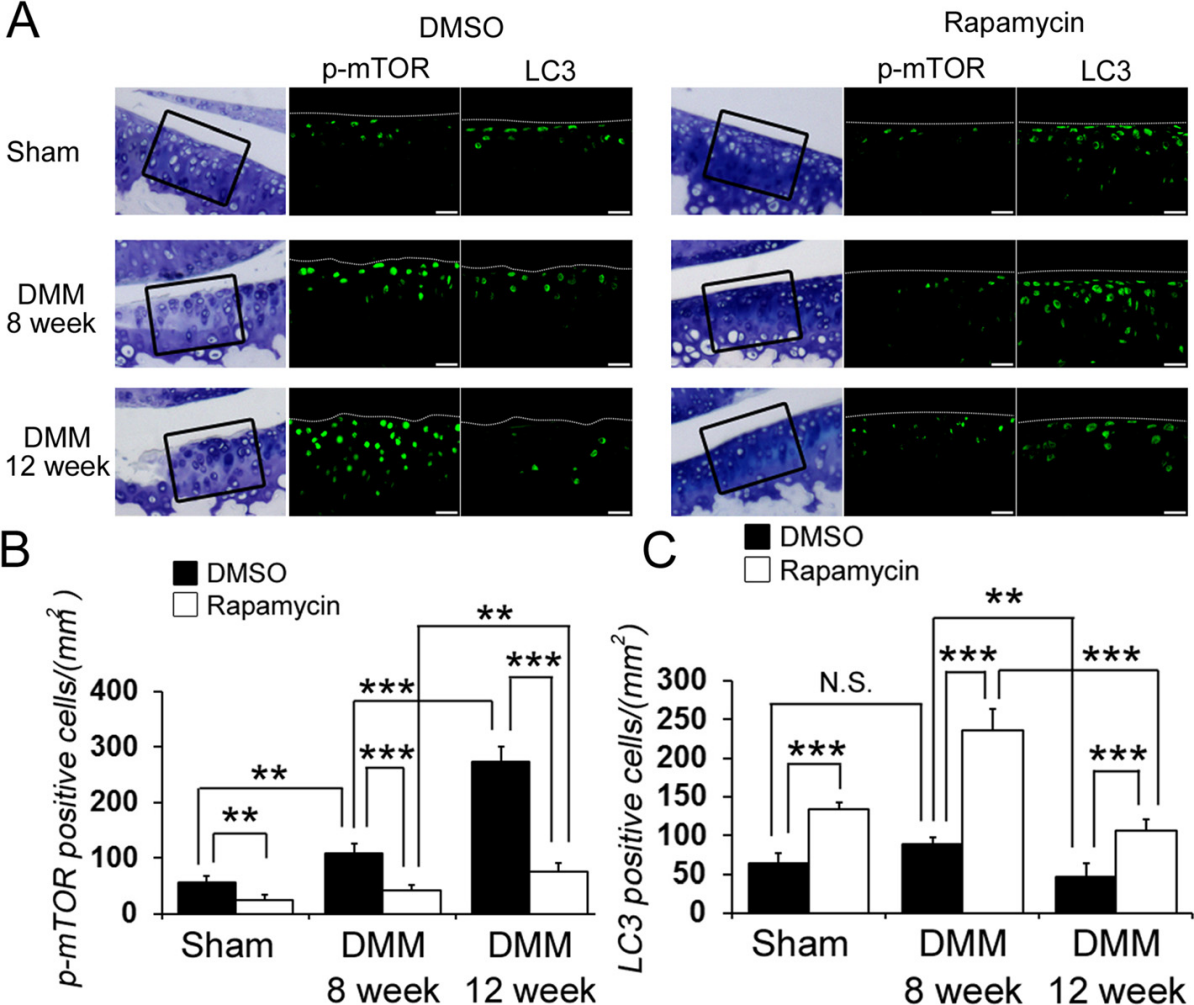
**The effect of rapamycin on phospho- mammalian target of rapamycin (p-mTOR) and light chain 3 (LC3) expression. (A)** Representative images of immunostaining for p-mTOR (green) and LC3 (green). Scale bar = 20 μm. **(B)** Quantification of p-mTOR-positive cells was calculated. Rapamycin treatment suppressed p-mTOR expression in the rapamycin-treated mice compared to those treated with dimethyl sulphoxid (DMSO) at both the 8- and 12-week time points (at 8 weeks DMSO 108.5 ± 13.3, rapamycin 43.0 ± 8.9 mm; at 12 weeks DMSO 273.8 ± 27.0, rapamycin 76.5 ± 14.7 mm). **(C)** Quantification of LC3-positive cells was calculated. **P <0.01, ***P <0.001. DMM, destabilization of the medial meniscus.

To determine whether the local intra-articular injection of rapamycin modulates mTOR and autophagy, the effects that rapamycin had on phospho-mTOR (p-mTOR) and LC3 expressions were examined. The expression of p-mTOR was increased in DMSO-treated mice at 8 and 12 weeks post DMM surgery compared to DMSO-treated mice undergoing the sham knee operation. Rapamycin treatment suppressed p-mTOR expression in these mice compared to those treated with DMSO at both the 8- and 12-week time points post DMM injury. The expression of p-mTOR in the rapamycin-treated mice was significantly increased at 12 weeks compared to 8 weeks after DMM injury (at 8 weeks DMSO 108.5 ± 13.3, rapamycin 43.0 ± 8.9; at 12 weeks DMSO 273.8 ± 27.0, rapamycin 76.5 ± 14.7) (Figure 2A, B). In contrast, there were more LC3-positive cells in the articular cartilage maintaining proteoglycan staining but fewer in the degenerated articular cartilage of the DMSO-treated mice after DMM surgery. An increase in LC3-positive cells was observed in the rapamycin-treated mice compared to those treated with DMSO at 8 and 12 weeks post DMM surgery. The number of LC3-positive cells in the rapamycin-treated mice decreased significantly at 12 weeks compared to 8 weeks post DMM injury (at 8 weeks DMSO 88.2 ± 13.1, rapamycin 236.0 ± 56.3; at 12 weeks DMSO 46.2 ± 12.8, rapamycin 106.3 ± 27.1) (Figure 2A, C).

### The intra-articular injection of rapamycin decreased VEGF expression in articular cartilage

**Figure 3:**
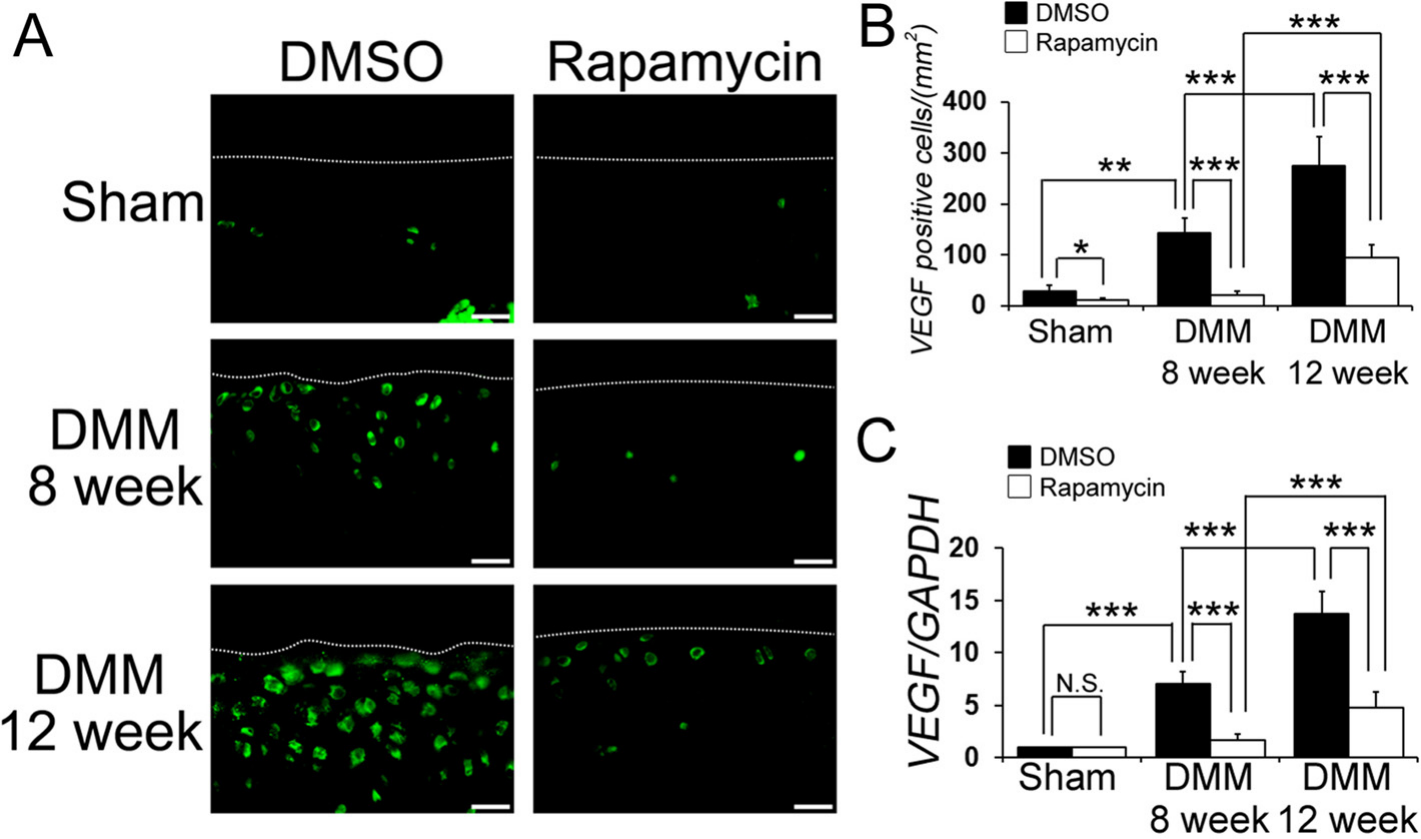
**The effect of rapamycin on vascular endothelial growth factor** (**VEGF) expression in the articular cartilage. (A)** Representative images of immunostaining for VEGF (green). Scale bar = 20 μm. **(B)** Quantification of VEGF-positive cells in the cartilage was calculated. **(C)** Graph indicating quantitative RT-PCR for VEGF in dimethyl sulphoxide (DMSO)- and rapamycin-treated mice at 8 and 12 weeks after destabilization of the medial meniscus (DMM) or sham surgery. *P <0.05, **P <0.01, ***P <0.001.

As it has been reported that the expression of VEGF is related to the development of OA changes [16], VEGF expression was examined with immunohistochemistry and qPCR (Figure 3). The number of VEGF-positive cells in the articular cartilage was significantly lower in the rapamycin group compared with the DMSO group at 8 and 12 week post DMM injury (at 8 weeks DMSO 143.8 ± 29.6, rapamycin 21.2 ± 8.4, *P* <0.001 for DMSO versus rapamcyin; at 12 weeks DMSO 275.5 ± 56.9, rapamycin 96.0 ± 27.8, *P* <0.001 for DMSO versus rapamcyin). The number of VEGF-positive cells in the rapamycin-treated mice increased at 12 weeks compared to 8 weeks post DMM injury (*P* <0.001 for DMSO at 8 weeks versus 12 weeks) (Figure 3A, B). Similarly, the expression of VEGF by qPCR revealed a significant increase in the DMSO-treated mice at 8 weeks after DMM surgery compared to sham knees (Figure 3C, *P* <0.001 relative to sham). Rapamycin treatment decreased VEGF expression when compared to the DMSO-treated mice at the 8 and 12 week time points post DMM injury (Figure 3C, *P* <0.001 at 8 and 12 weeks), suggesting that the local intra-articular injection of rapamycin reduced articular cartilage damage, at least in part, through a reduction in VEGF expression.

### The local intra-articular injection of rapamycin decreased CoL10A1 and MMP13 expression

**Figure 4:**
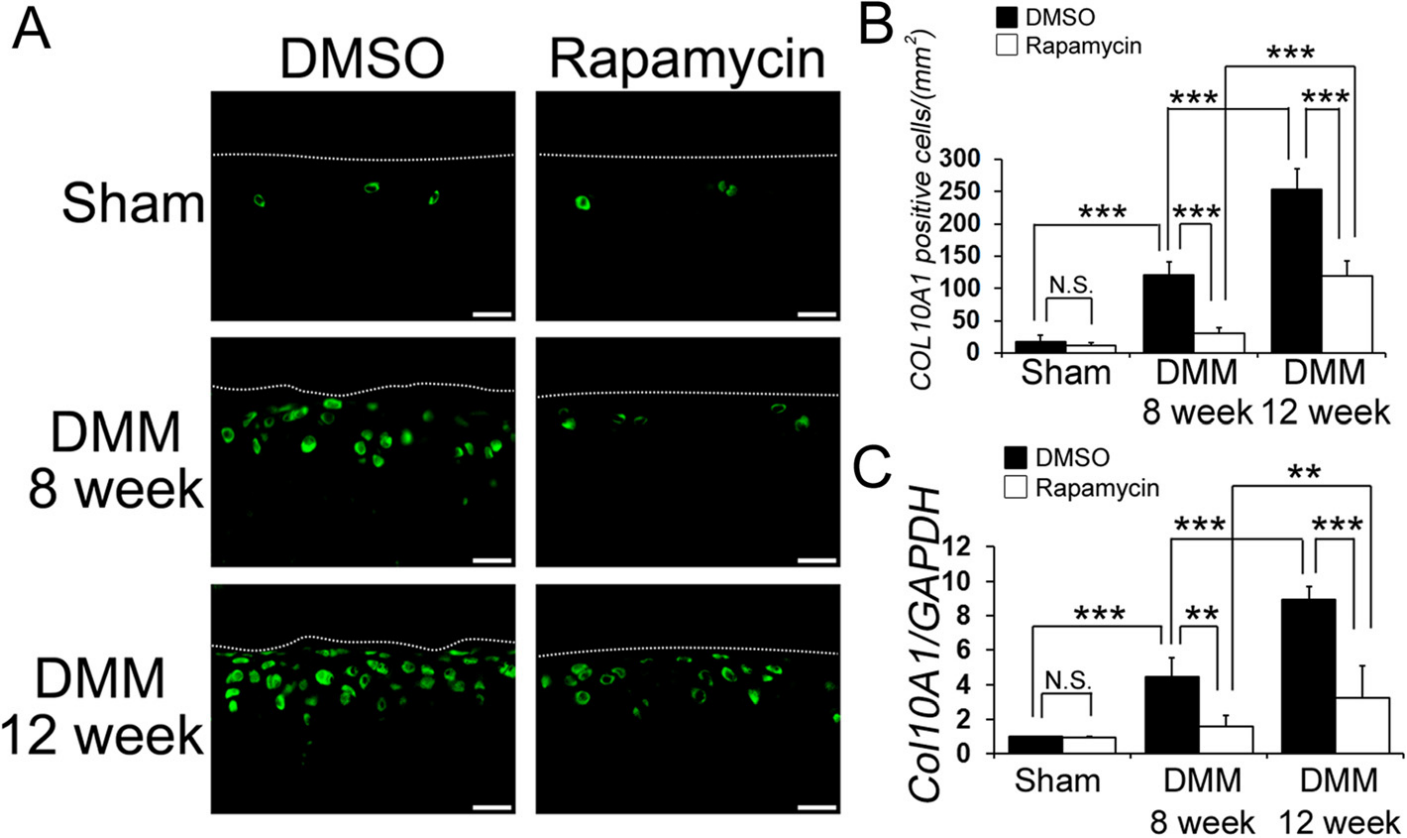
**The effect of rapamycin on collagen, type X alpha 1 (COL10A1) expression in articular cartilage. (A)** Representative images of immunostaining for COL10A1 (green). Scale bar = 20 μm. **(B)** Quantification of COL10A1-positive cells in the cartilage was calculated. **(C)** Graph indicating quantitative RT-PCR for COL10A1 in dimethyl sulphoxide (DMSO)- and rapamycin-treated mice 8 and 12 weeks after destabilization of the medial meniscus (DMM) injury. ***P <0.001, **P <0.01.

**Figure 5:**
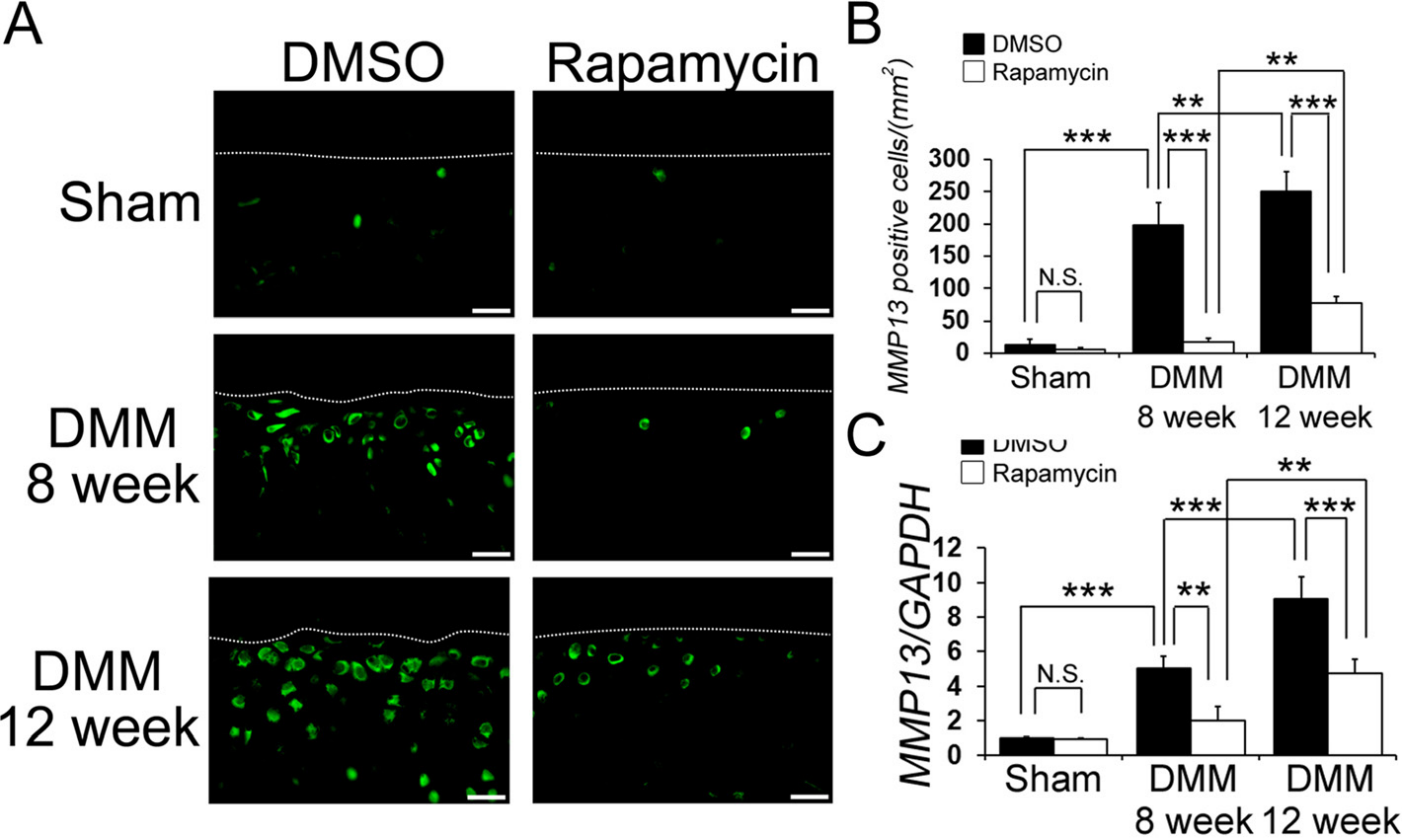
**The effect of rapamycin on matrix metallopeptidase 13 (MMP13) expression in articular cartilage. (A)** Representative images of immunostaining for MMP13 (green). Scale bar = 20 μm. **(B)** Quantification of MMP13-positive cells in the cartilage was calculated. **(C)** Graph indicating quantitative RT-PCR for MMP13 in dimethyl sulphoxide (DMSO)- and rapamycin-treated mice 8 and 12 weeks after destabilization of the medial meniscus (DMM) injury. ***P <0.001, **P <0.01.

To investigate the mechanism behind rapamycin’s ability to prevent experimental OA, subsequent experimentation focused on chondrocyte hypertrophy and consequently the expression of COL10A1 and MMP13 (hypertrophic chondrocyte markers) was examined by immunohistochemistry and qPCR (Figures 4, 5). The number of CoL10A1-positive cells in the articular cartilage was significantly lower in the rapamycin group when compared to the DMSO group at 8 and 12 weeks post DMM injury (at 8 weeks DMSO 88.2 ± 13.1, rapamycin 236.0 ± 56.3, *P* <0.001 for DMSO versus rapamcyin; at 12 weeks DMSO 46.2 ± 12.8, rapamycin 106.3 ± 27.1, *P* <0.001 for DMSO versus rapamcyin). The number of CoL10A1-positive cells in the rapamycin-treated mice increased at 12 weeks compared to 8 weeks post DMM injury (*P* <0.001 for DMSO at 8 weeks versus 12 weeks) (Figure 4A, B). Similarly the expression of COL10A1, as measured by qPCR, was increased in the DMSO-treated mice at 8 and 12 weeks after DMM surgery compared to the sham knees (Figure 4C: *P* = 0.006 at 8 weeks, *P* <0.001 at 12 weeks, respectively). Also intra-articular injection of rapamycin significantly decreased the expression of COL10A1 compared to the DMSO-treated mice at 8 and 12 weeks after DMM surgery (Figure 4C: *P* = 0.001at 8 weeks, *P* <0.001 at 12 weeks, respectively).

The number of MMP13-positive cells in the cartilage was significantly lower in the rapamycin group compared with the DMSO group at 8 and 12 weeks post DMM injury (at 8 weeks DMSO 198.2 ± 34.6, rapamycin 18.0 ± 5.7, *P* <0.001 for DMSO versus rapamcyin; at 12 weeks DMSO 249.8 ± 31.1, rapamycin 77.8 ± 10.0, *P* <0.001 for DMSO versus rapamcyin). The number of MMP13-positive cells in the rapamycin-treated mice increased at 12 weeks compared to 8 weeks post DMM injury (*P* <0.01 for DMSO at 8 weeks versus 12 weeks) (Figure 5A, B). Rapamycin treatment reduced MMP13 expression, as measured by qPCR, at 8 and 12 weeks after DMM surgery compared to the MMP13 expression in the DMSO-treated mice (Figure 5C, *P* = 0.026 at 8 weeks, *P* <0.001 at 12 weeks). These results suggest that mTOR inhibition with rapamycin decreases hypertrophic change in articular cartilage after DMM surgery.

## Discussion

The results of the current study are the first to demonstrate that articular cartilage degeneration occurring after DMM surgery correlates with increased in mTOR expression, and consequently local intra-articular injection of rapamycin reduced mTOR expression, which delayed articular cartilage degradation. Recently, systemic injection of rapamycin has been shown to reduce the severity of OA in an experimental murine model [9]. The systemic use of rapamycin in the clinical setting is associated with many side effects, such as weight loss, skin rashes, delayed wound healing, or diarrhea, which can eventually lead to rapamycin withdrawal [12]. In this study, these side effects were not observed after local intra-articular injection of rapamycin. The local intra-articular injection of rapamycin might be more appropriate for clinical use than systemic administration.

In this study, we demonstrated that after surgically inducing joint instability, chondrocytes in the articular cartilage displayed increased mTOR expression during the progression of OA, suggesting that the activation of mTOR leads to articular cartilage degeneration. Furthermore, the inhibition of mTOR with rapamycin promoted a significant delay in the progression of OA changes, as demonstrated histologically by staining the AC for proteoglycan content.

**Figure 6:**
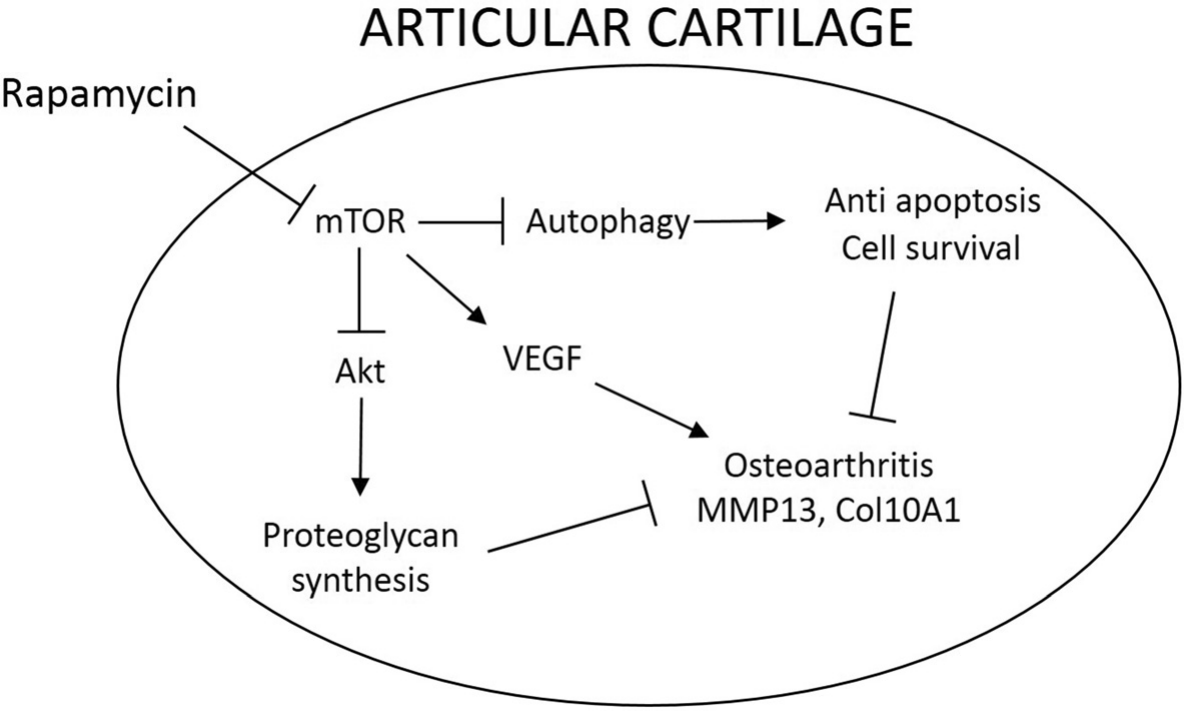
**Proposed schematic model showing how rapamycin modulates osteoarthritis (OA) in our animal model.** There are three potential mechanisms by which rapamycin has a beneficial effect on OA (autophagy, vascular endothelial growth factor (VEGF), and the Akt pathway). mTOR, mammalian target of rapamycin; MMP13, matrix metallopeptidase 13; COL10A1, collagen, type X alpha 1.

Recent studies have demonstrated that mTOR inhibition by rapamycin triggers a negative feedback loop, resulting in the activation of Akt signaling [17,18]. In adult articular cartilage, phosphoinositide 3 (PI-3) kinase-Akt signaling promotes matrix synthesis as well as the survival of chondrocytes. Activation of Akt in human articular chondrocytes significantly increase proteoglycan synthesis and type II collagen expression [19-21] (Figure 6). Another important function of the mTOR signaling pathway is the regulation of autophagy, a process in which the cell degrades damaged or excess cellular components, ranging from individual proteins and protein aggregates to whole organelles, through the use of the cell lysosomal machinery. A previous *in vitro* study indicated that autophagy activation by 10 μM rapamycin regulated a change in the expression of OA-related genes through the modulation of apoptosis and reactive oxygen species (ROS) in human chondrocytes [8]. Similarly, during the development of OA, autophagy increased as an adaptive response to protect the cells from various stresses, while in severely damaged articular cartilage, autophagy was reduced [8]. The present study demonstrated that LC3-positive cells resided in healthy articular cartilage maintaining proteoglycan staining, and LC3-expressing cells decreased in degenerating articular cartilage after DMM surgery. Thus, it is plausible that articular cartilage degradation after surgical induction of OA is partially due to insufficient autophagy. In support of this hypothesis, our results also indicate that the beneficial effect of local intra-articular rapamycin treatment on OA-induced cartilage damage correlates with an increase in LC3-expressing cells (Figure 6).

Subsequently, we focused on the potential role of angiogenesis in modulating articular cartilage degeneration after OA and whether the beneficial effect of rapamycin can be explained by modulation of angiogenesis. Accumulating evidence indicates that joints affected by OA contain increased levels of VEGF in their articular cartilage and synovial fluid as well as several cytokines that stimulate VEGF production [16,22,23]. In a recent publication we have demonstrated that the intra-articular injection of stem cells transduced with sFLT1, an antagonist of VEGF, could reduce angiogenesis and improve the regeneration of articular cartilage without osteophyte development in a rat model of OA [24]. Interestingly, mTOR activation stimulates VEGF [25,26], whereas rapamycin treatment reduces VEGF expression, which leads to the suppression of endothelial cell proliferation, survival, and migration [27]. In the current study, local intra-articular injection of rapamycin decreased the expression of VEGF after DMM when compared with the DMSO-treated mice, suggesting that decreased VEGF expression might play a role in the beneficial effect of rapamycin on articular cartilage after OA (Figure 6).

To further examine the beneficial effect of rapamycin on articular cartilage after OA, we investigated chondrocyte hypertrophy. Chondrocyte hypertrophic-like changes also contribute to the progression of early and late stages of OA [28]. The induction of hypertrophic-like changes in healthy human chondrocytes causes calcification of the extracellular matrix [29,30]. Recently, hypertrophic differentiation of chondrocytes has also been reported to promote angiogenesis [31]; hence, the inhibition of chondrocyte hypertrophic-like alterations could be a therapeutic target to block the progression of OA [28]. mTOR is associated with the development of hypertrophic changes [32-34], and mTOR inhibition by rapamycin decreases chondrocyte hypertrophy in the growth plate [35,36]. The expression of COL10A1 and MMP13 are the most widely used markers for identifying hypertrophic chondrocytes [37-39]. In the current study, rapamycin treatment was found to reduce the expression of COL10A1 and MMP13 in comparison to mice treated with DMSO after DMM surgery. However, in the rapamycin-treated mice, the expression of COL10A1 and MMP13 at 12 weeks were increased compared to those at 8 weeks. These findings suggest that the intra-articular injection of rapamycin reduced the hypertrophic changes in the articular cartilage in this experimental model of OA, which contributed to a delay in OA progression.

There are some limitations in this study. First, the optimal dosage and frequency of rapamycin might be different in mice and humans, although the results suggest that local intra-articular injection of rapamycin delayed articular cartilage degradation. We also need to examine the effect of intra-articular injection of rapamycin at a lower dosage and frequency in larger animal models, before we apply the injection of rapamycin in a clinical setting. Second, experimental OA was induced by DMM. Lesions in the DMM model progressed from mild to moderate OA compared to the anterior cruciate ligament transection model. Therefore, experimental OA induced by DMM differs from the primary OA observed in humans. In addition, young mice were used in this study, and although young mice have been widely used for this type of study, the regenerative capacity in young animals is most likely superior to that of aged animals, which could obscure the process of cartilage degeneration. In future studies, aged and large animal models should be utilized to test the effects of rapamycin treatment on articular cartilage in primary OA.

## Conclusion

In conclusion, our results demonstrated that the intra-articular injection of rapamycin reduce mTOR expression, which leads to a delay in cartilage degradation after surgically inducing joint instability. Activation of LC3 (an autophagy marker) in the OA-induced chondrocytes as well as reduction in VEGF, COL10A1, and MMP13 expression was also observed in the rapamycin-treated knees. Our observations suggest that local intra-articular injection of rapamycin may represent a strategy to prevent the development of articular cartilage damage, while limiting the side effect of systemic delivery of rapamycin. Further studies that explore mTOR inhibition will provide novel insights into the pathophysiology of OA and could lead to the establishment of new therapeutic approaches for slowing the progression of OA.

## Abbreviations

COL10A1: collagen, type X alpha 1
DMM: destabilization of the medial meniscus
DMSO: dissolved in dimethyl sulphoxide
ECM: extracellular matrix
ER: endoplasmic reticulum
LC3: light chain 3
MMP13: matrix metallopeptidase 13
mTOR: mammalian target of rapamycin
OA: osteoarthritis
PBS: phosphate-buffered saline
PI-3 kinase: phosphoinositide 3 kinase
p-mTOR: phospho- mammalian target of rapamycin
qPCR: quantitative RT-PCR
ROS: reactive oxygen species
VEGF: vascular endothelial growth factor
